# Supplementary material for: Effect of THz Waves of Different Orientations on K+ Permeation Efficiency in the KcsA Channel
Source: Int J Mol Sci. 2023 Dec 28;25(1):429. doi: 10.3390/ijms25010429 (PMC10779155; doi:10.3390/ijms25010429)
Supplement: Supplementary file 1 [file ijms-25-00429-s001.zip › ijms-2762964-supplementary.pdf]

Supporting Information

Contents

S1. Simulation system preparation
S2. Simulation methods
S3. Setting of charge difference parameters
S4. High-frequency electromagnetic fields act on the KcsA channel
S5. Ion flux under different electric field strength
S6. Choice of electric field frequency
S7. Viewing the structure of the SF region in the VMD
S8. Distribution of φ dihedral angles of key residues
References

S1. Simulation system preparation

The simulated systems selected the KcsA (PDB:5VK6) channel which has an open inner helix bundle gate and an activated selectivity filter in Fig.1. This channel was embedded in a patch of a palmitoyl-oleoyl-phosphatidylcholine (POPC) membrane containing 134 lipid molecules, which was then hydrated by 10359 TIP3P water molecules. KCl (0.3M) was also added to neutralize the system, resulting in a simulation system of 80.2×80.2×94.4 $Å^3$.

All molecular dynamics (MD) simulations were performed using GROMACS software version 2019.3 with CHARMM36 force field. Periodic boundary conditions were applied in the XYZ direction. PME was used to treat electrostatic interactions exceeding the 1nm cut-off and set the cutoff of vdW interactions to 1nm. The LINCS constraint algorithm was used to reset bonds after an unconstrained update of 2 fs. The pressure and temperature were held at 1 atm and 300 K by the semi-isotropic Parrinello-Rahman barostat and the v-rescale thermostat respectively. The systems performed a steepest descent energy minimization and then equilibrated until the system stabilized. In the 5 ns of the NVT and 15 ns of NPT equilibration simulations, the heavy atoms of protein were restrained with a force constant of 1000 kJ mol^{-1} nm^{-2} to their starting positions. Lipids, ions, and water were allowed to move freely during equilibration.

S2. Simulation methods

S2.1. The ion imbalance method

The Computational Electrophysiology (CompEL) method in GROMACS is used to simulate the ion flux through the KcsA channel[1], driven by transmembrane potentials (TM). We duplicated the well-equilibrated single-layered system in the direction of the membrane normal (Z-axis) to prepare a double-layered system for CompEL simulations. The transmembrane potential is caused by an ion imbalance between the two sides of the membrane, which is also called the ion imbalance (IIMB) method[[1,2]]. The .mdp file was set to force the small charge imbalance Δq and we set the ion imbalance between the compartments as 4e.

S2.2. The combined method

At present, there are mainly two methods to construct transmembrane potential in molecular simulation, one is the IIMB method mentioned above, and the other is the constant electric field (CEF) method[3-5]. In the CEF method, the (TM) potential is simulated by adding an applied electric field along the membrane's normal direction. The TM voltage ΔV can be calculated by $\Delta V = \vec{E} \cdot L$, where L is the length of the simulation box, $\vec{E}$ is the strength of the electric field, and the charged particles are subjected to a constant additional force $\vec{F} = q_i\vec{E}$.

In the combined model, the TM potential is constructed by the IIMB method, and

when the number of potassium (K) ions on each side of the membrane deviates from the reference count, the forced exchange between K^+ ions and water was carried out, which was similar to the ion pump in real cells. The CEF method is used to apply an applied electric field to the whole simulation system, so as to simulate the influence of the THz electric field on KcsA K^+ ion channel.

The simulation results of the IIMB method were used as the reference for the simulation without the applied electric field, and the CEF-IIMB combined method was used for the simulation of the terahertz electric field with different frequencies acting on the KcsA channel.

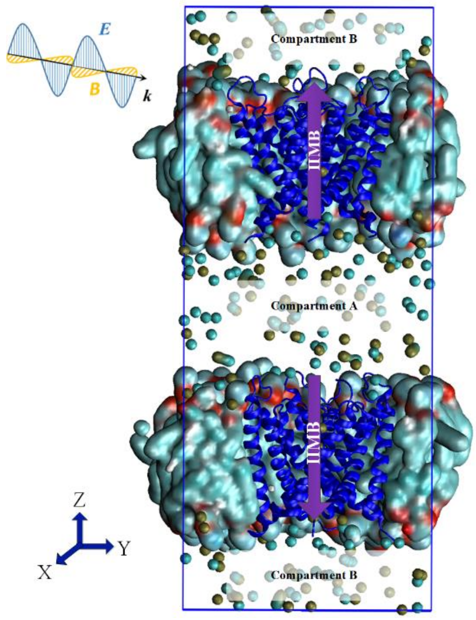

Figure S1 | The CEF-IIMB combined model. The simulation system consists of two membranes, each including open KcsA, surrounded by water and ions (the yellow balls are K+, the blue balls are Cl-). The transmembrane potential is generated by sustaining a small charge imbalance Δq between compartments A and B by the IIMB method. The CEF method is used to simulate the external THz-EM field.

S3. Setting of the charge difference parameter

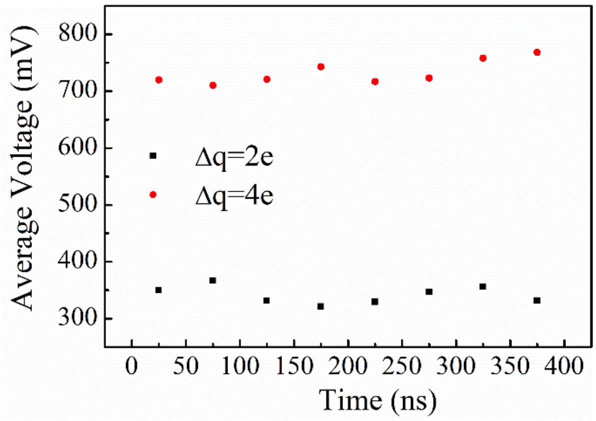

Figure S2 | Membrane voltage at different charge differences.

The transmembrane potential in CompEL is achieved by sustaining a small imbalance of charges Δq across the membrane. Fig.S2 shows the average voltage of several independent simulation results every 50 ns when the simulation system was set with different charge differences. When Δq=2e, the voltage fluctuates around 330mV, and when Δq=4e, the voltage fluctuates around 720 mV. Fig.S2 shows the average voltage of several independent simulation results every 50 ns when the simulation system is set with different charge differences. When Δq=2e, the voltage fluctuates around 330 mV, and when Δq=4e, the voltage fluctuates around 720 mV. Fig. S3 shows the ion flux at different charge differences. The average ion flux curve of Δq=2e is obtained by independently simulating 20 groups with a total time of 8 μs, but the ion flux curve has not yet converged. The transmembrane voltage when Δq=4e is relatively high, but compared to the case of Δq=2e, there is a more stable ion current. At the same time, the effect of the external THz electric field on the ion flux can be seen more clearly.

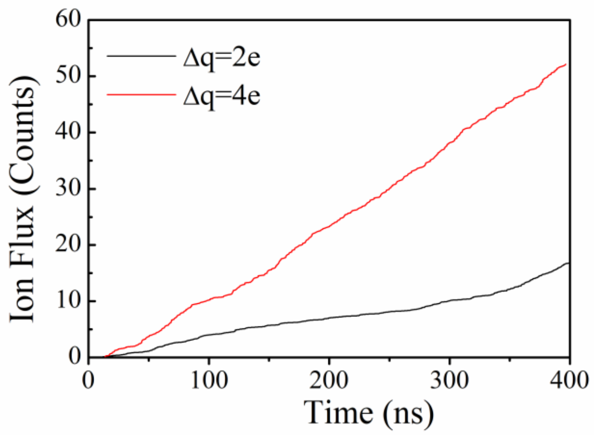

Figure S3 | Ion flux at different charge differences.

S4. High-frequency electromagnetic fields act on KcsA channels

The wavelength of 1THz electromagnetic wave in free space is

$$\lambda = \frac{c}{f} = \frac{3 \times 10^8}{1 \times 10^{12}} = 3 \times 10^{-4} m = 300 \mu m$$

and the size of the single box we simulated is 80.2×80.2×94.4 Å^3. As the wave of terahertz electromagnetic wave is longer than the size of the simulated region, quasi-stable approximation is adopted in the simulation process.

For the plane electromagnetic wave with electric field intensity $E(t)$ and magnetic field intensity $H(t)$ in free space, the electric field force f_e and magnetic field force f_m subjected to charged particles with charge q and velocity v are respectively:

$$f_e = qE(t)$$

$$f_m = qv\mu_0 H(t)$$

Then the ratio of the magnetic field force to the electric field force on the ion is:

$$\frac{f_m}{f_e} = v\mu_0 \frac{H(t)}{E(t)} = v\mu_0 \sqrt{\frac{\varepsilon_0}{\mu_0}} = v\sqrt{\mu_0 \varepsilon_0} = \frac{v}{c}$$

Because charged particles travel much less than the speed of light, the electric field force on the charged particles is much greater than the magnetic field force. Therefore, in the simulation, the influence of the high-frequency magnetic field can be ignored and only the influence of the high-frequency electric field can be considered. Therefore, for the external sinusoidal alternating electric field with amplitude of electric field intensity E_0 and frequency $2\pi\omega$ Hertz, the following expression can be adopted:

$$E(t) = E_0 \cos\left[\omega(t - t_0)\right]$$

S5. Ion flux under different electric field strength

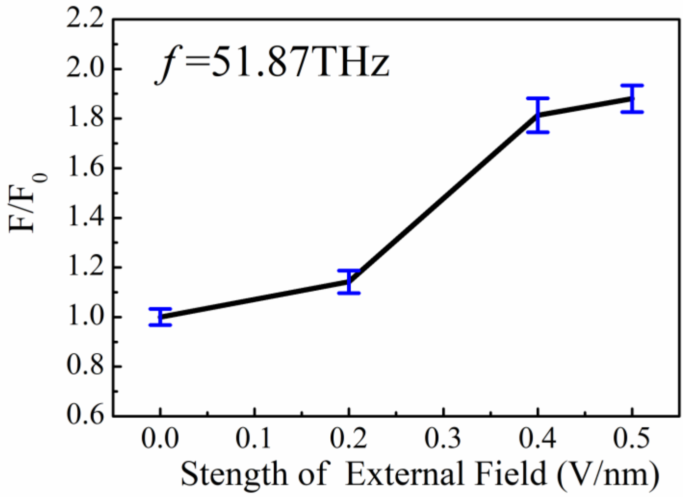

Figure S4 | The influence of electric field amplitude on ion flux. The ion flux is normalized in the situation without the external field (F_0).

We simulated the dynamics for the four cases where the frequency of the external electric field was 51.87 THz and the amplitude was 0.2 V/nm, 0.4 V/nm, 0.5 V/nm, and 0.6 V/nm, respectively. The direction of incidence of these electric fields is along the z-axis. Similar to the simulation process without the THz electric field, we performed 8 independent simulations with different amplitudes of the 51.87 THz electric field for a total time of 3.2 μs and averaged the ion fluxes obtained from different amplitudes simulation. The ion fluxes under electric fields with different amplitudes were normalized to the ion flux without the external electric field, shown in Fig. S4. The ion flux with an amplitude of 0.2 V/nm is close to the ion flux without the THz field, which is 1.14 times that without the THz field, while the ion flux with an amplitude of 0.4 V/nm is 1.8 times that without the THz field. The ion flux at a value of 0.5 V/nm is close to the ion flux at 0.4 V/nm, which is 1.87 times that without the external THz field. We have also simulated the case where the amplitude is 0.6 V/nm, but it may be because the field strength exceeds the threshold, the simulation system always collapsed around 200 ns. So we finally chose 0.4 V/nm as the amplitude of the external electric field when comparing the influence of frequency on ion flux.

S6. Choice of electric field frequency

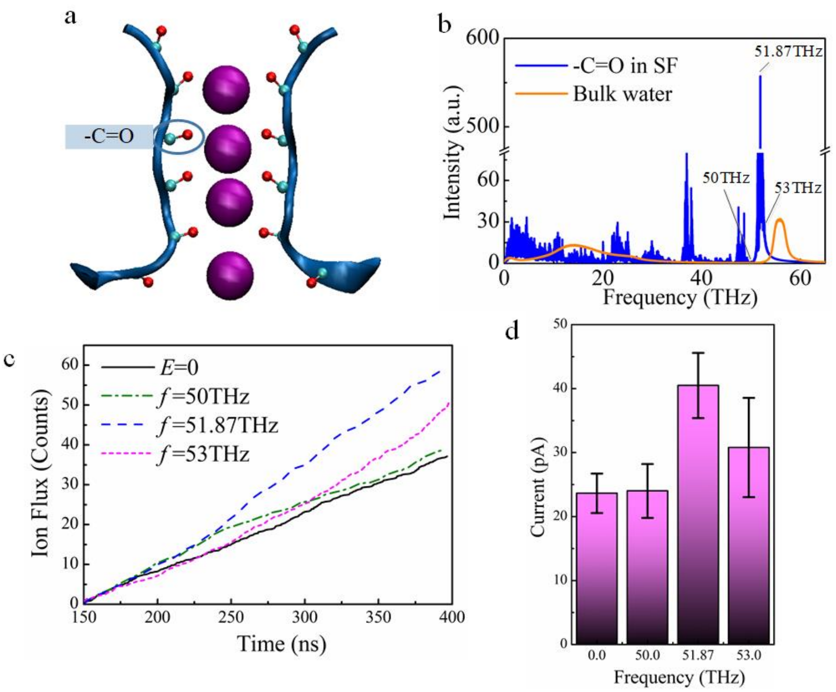

Figure S5 | THz electric field increases the ion flux. (a) The oxygen atoms in -C=O groups of the SF region are tightly bound to K^+. (b) Comparison between the absorption of the -C=O groups in the SF (blue curve) and that of the bulk water (orange curve). (c) Changes of ion flux caused by THz electric field with different frequencies. (d) The effect of different frequencies on the current[6].

The selectivity filter is a key region for K^+ permeation in K^+ channels, with the S_0-S_4 sites on the SF being the most important. In a previous paper, we analyzed the infrared absorption spectra of carbonyl oxygen atoms in the SF region and found that the K^+ current in the channel was highest at the applied electric field frequency of 51.87 THz [6].

S7. Viewing the structure of the SF region in the VMD

We observe the structure of the SF during transport. In addition to the common direct Coulomb knock-on mechanism, we find that the structure of the SF is more prone to distortion when the incident angle of the applied electric field is θ=90°. When this happens, there are usually only two K^+ in the SF, and the K^+ always stay in the SF in the state of 'S3, S4' or 'S3, S2'. K^+ takes a longer time to pass through the SF region. In this case, the deformation of the SF region of the KcsA channel can be clearly observed in the VMD.

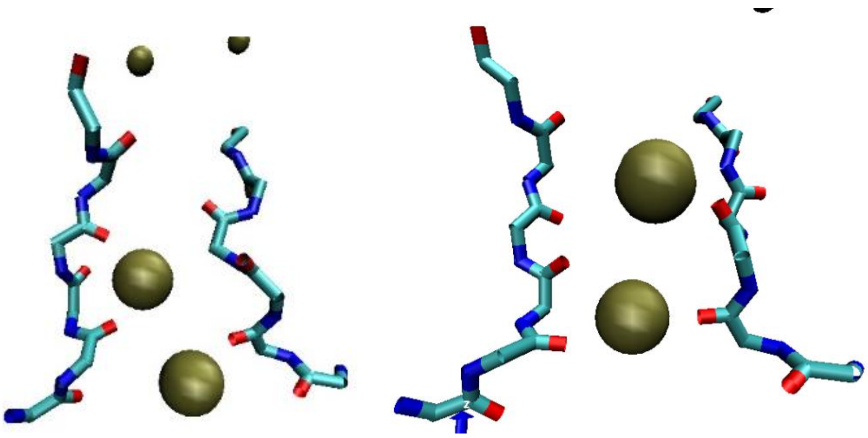

Figure S6 | When θ = 90°, a distortion of the SF structure is observed in the VMD.

S8. Distribution of φ dihedral angles of key residues

KcsA is a tetrameric structure that is cross-symmetric about the central pore, so there are four symmetric residues at each site. The figure shows the distribution of dihedral angles of the symmetric residues at each site.

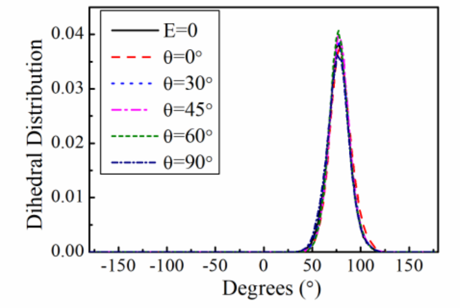

THR75_chain1

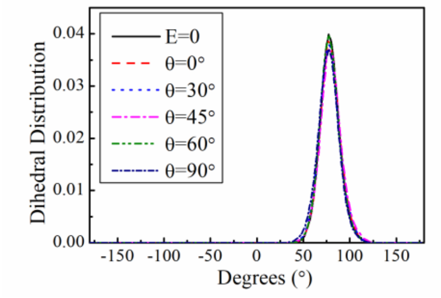

THR75_chain2

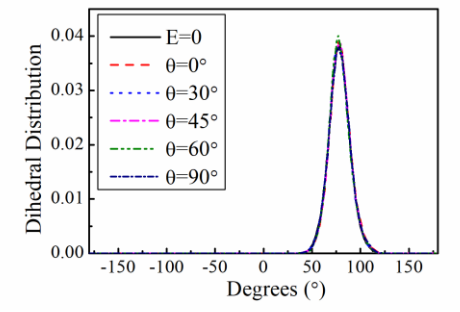

THR75_chain3

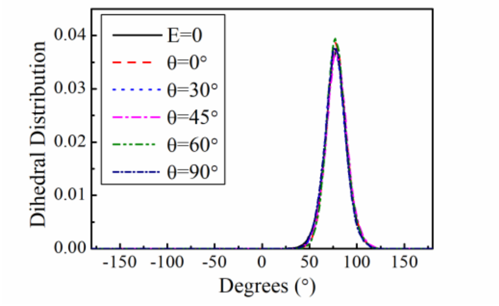

THR75_chain4

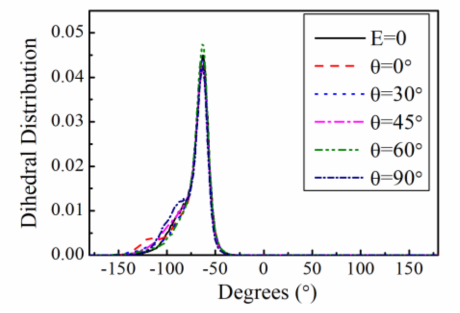

VAL76_chain1

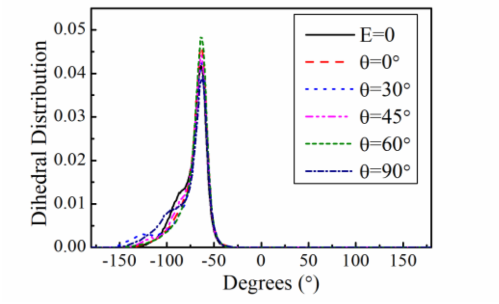

VAL76_chain2

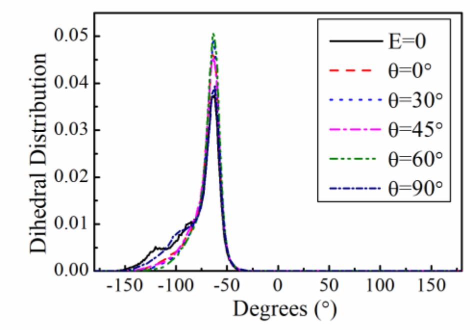

VAL76_chain3

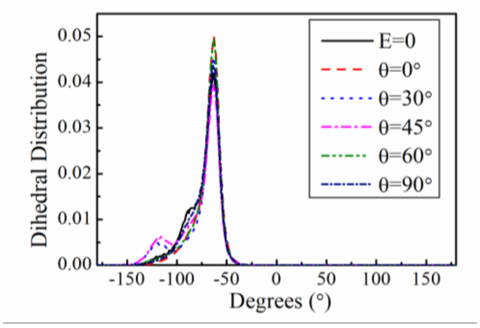

VAL76_chain4

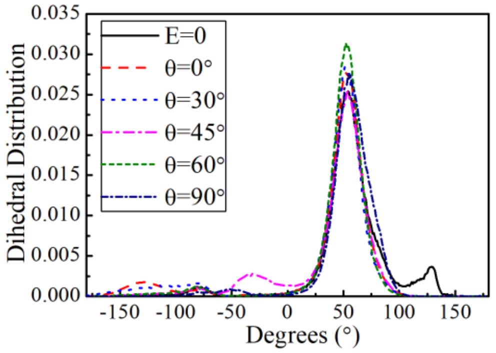

GLY77_chain1

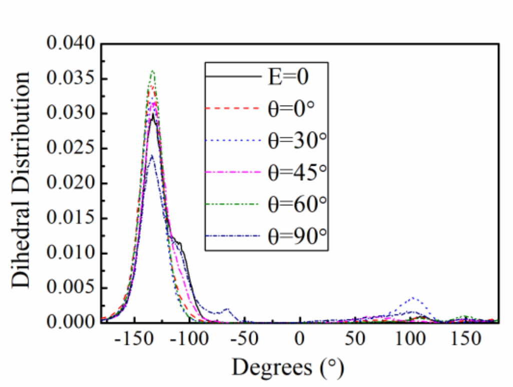

GLY77_chain2

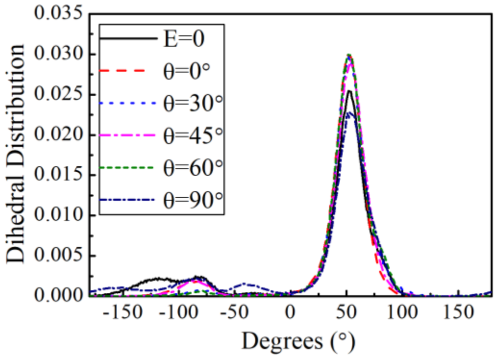

GLY77_chain3

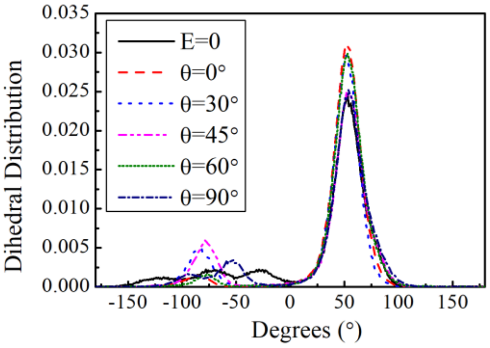

GLY77_chain4

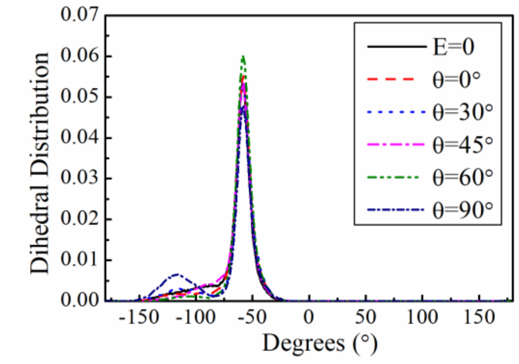

TYR78_chain1

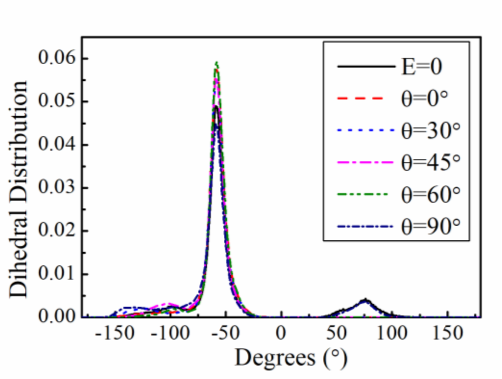

TYR78_chain2

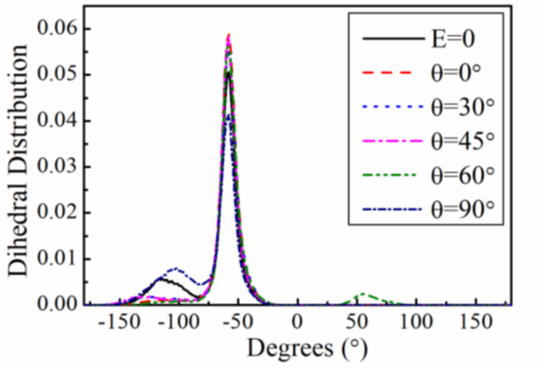

TYR78_chain3

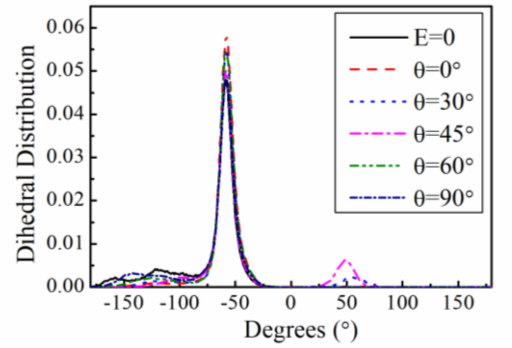

TYR78_chain4

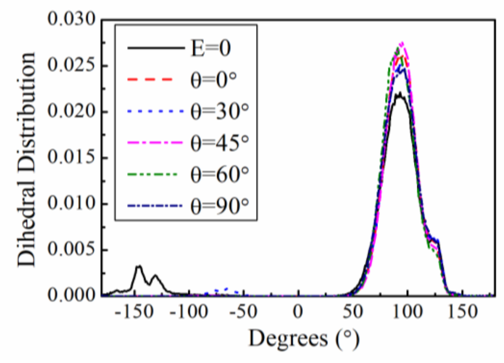

GLY79_chain1

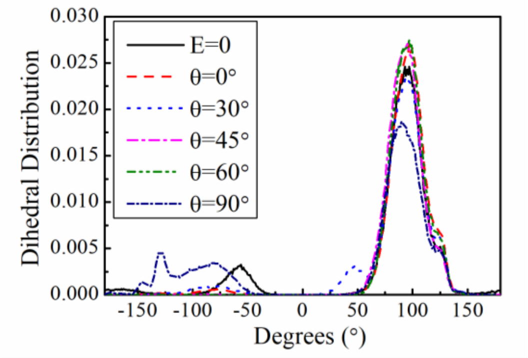

GLY79_chain2

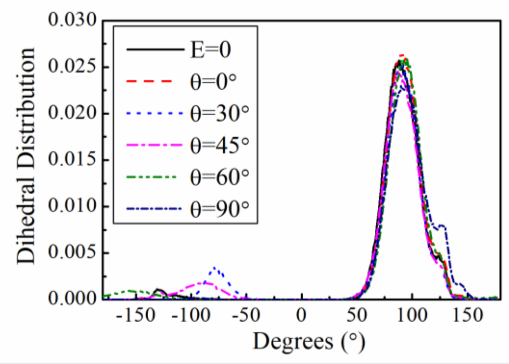

GLY79_chain3

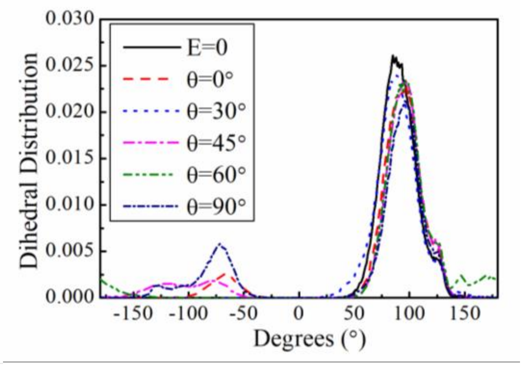

GLY79_chain4

References

(1) Kutzner, C.; Grubmüller, H.; de Groot, B. L.; Zachariae, U. Computational Electrophysiology: The Molecular Dynamics of Ion Channel Permeation and Selectivity in Atomistic Detail. Biophys. J. 2011, 101, 809−817.

(2) Sachs, J. N., P. S. Crozier, and T. B.Woolf. Atomistic Simulations of Biologically Realistic Transmembrane Potential Gradients. J. Chem. Phys. 2004, 121, 10847–10851.

(3) Roux, B. The Membrane Potential and its Representation by a Constant Electric Field in Computer Simulations. Biophys. J. 2008, 95, 4205-4216.

(4) Delemotte, L.; Dehez, F.; Treptow, W.; Tarek, M. J. Modeling Membranes under a Transmembrane Potential. Phys. Chem. B. 2008, 112, 5547−5550.

(5) Gumbart, J.; Khalili-Araghi, F.; Sotomayor, M.; Roux, B. Constant Electric

Field Simulations of the Membrane Potential Illustrated with Simple Systems. Biochim. Biophys. Acta, Biomembr. 2012, 1818, 294−302.
(6) Wang Y.; Wang H. Regulation of ion permeation of the KcsA channel by applied midinfrared field. IJMS. 2023,24(1), 556.
